# Conditional knockout of UBC13 produces disturbances in gait and spontaneous locomotion and exploration in mice

**DOI:** 10.1038/s41598-019-40714-3

**Published:** 2019-03-13

**Authors:** David F. Wozniak, Pamela Valnegri, Joshua T. Dearborn, Stephen C. Fowler, Azad Bonni

**Affiliations:** 1 Department of Psychiatry, Washington University School of Medicine, St. Louis, MO 63110 USA; 2 Department of Neuroscience, Washington University School of Medicine, St. Louis, MO 63110 USA; 3 Department of Internal Medicine, Washington University School of Medicine, St. Louis, MO 63110 USA; 4 Taylor Family Institute for Innovative Psychiatric Research, Washington University School of Medicine, St. Louis, MO 63110 USA; 5 Department of Pharmacology and Toxicology, University of Kansas, Lawrence, KS 66045 USA

## Abstract

Here we have characterized the functional impairments resulting from conditional knockout of the ubiquitin-conjugating E2 enzyme (UBC13) in rodent cerebellar granule neurons, which greatly increases the parallel fiber presynaptic boutons and functional parallel fiber/Purkinje cell synapses. We report that conditional UBC13 knockout mice exhibit reliable deficits on several gait-related variables when their velocity of ambulation is tightly controlled by a moving treadmill and by restricting space for movement. Selected gait parameters and movement patterns related to spontaneous exploration in an open field may also be affected in conditional UBC13 knockout mice. Analysis of open-field data as a function of test session half using force-plate actometer instrumentation suggest that conditional UBC13 knockout mice have alterations in emotionality, possibly affecting gait and movement variables. These findings suggest that conditional UBC13 knockout mice represent a valuable platform for assessing the effects of disturbances in cerebellar granule cell circuitry on gait and other aspects of locomotion. Also, the possibility that psychological factors such as altered emotionality may impact gait and movement patterns in these mice suggest that these mice may provide a useful model for evaluating analogous behavioral impairments in autism spectrum disorders and other neurodevelopmental syndromes associated with deregulation of ubiquitin signaling.

## Introduction

Deregulation of ubiquitin signaling is believed to contribute to the pathogenesis of neurodevelopmental disabilities such as autism spectrum disorders (ASD) and Angelman syndrome^1–3^. However, the functional effects of disturbances in autism-linked ubiquitin signaling in brain development remain to be fully characterized. Previously, we reported that *in vivo* knockdown or conditional knockouts of the autism-linked E3 ubiquitin ligase RNF8 or associated ubiquitin-conjugating E2 enzyme UBC13 in rodent cerebellar granule neurons resulted in enhanced numbers of parallel fiber presynaptic boutons and functional parallel fiber/Purkinje cell synapses^4^. In that study, we also described compromised cerebellar-dependent learning as indexed by delayed eyeblink conditioning to be a component of the behavioral phenotype of RNF8 or conditional UBC13 knockout mice. Although we have established this form of classical conditioning is impaired in RNF8 or conditional UBC13 knockout mice, we reasoned that there might be other behavioral deficits present in these mice, as well, due to cerebellar dysfunction. Considering impairment of synaptogenesis in the cerebellar cortex in conditional UBC13 knockout mice, and that the cerebellum plays a critical role in motor coordination^5,6^, provides justification for carefully studying disturbances in gait and movement patterns in these mice.

It was noted in earlier studies that the measurement of gait was complicated by the fact that animals including humans ambulate at different speeds, which may affect the values of relevant variables used to characterize it^7,8^. More recently, discussion of the appropriate use of technological advancements in the instrumentation used to evaluate gait and movement disturbances in rodent models of human disorders, including analysis of digital footprints, has reaffirmed the role of velocity in affecting gait metrics^9^. For example, analysis of the performance of wild type mice on the CatWalk gait analysis system (Noldus Inc, NE) showed that over 90% of the output variables associated with this technique are dependent on the speed of ambulation^10^. Use of the DigiGait imaging system (Mouse Specifics Inc, MA) provides control for this variable by having rodents ambulate on a treadmill where belt speed can be specified and altered. Use of this methodology helps to keep the speed of ambulation constant across groups so that various gait and movement indices may be compared without fluctuations in speed potentially confounding the data. However, rodents are not usually forced to move around their environment at invariant speeds, so performance on the DigiGait procedure might not reflect naturally-occurring motor behavior. Therefore, to provide full characterization of gait in rodents, it is important to include analyses that control for speed of ambulation as well as include quantification of spontaneously-occurring movements as rodents move about their environment. The CatWalk and force-plate actometer (FPA) instrumentation systems may be used to for this latter purpose. Although both techniques provide data on spontaneously-occurring ambulation without imposing a specified speed of movement, the analysis used with the CatWalk is focused more on gait-related metrics. In contrast, the FPA procedure also involves measurement of some standard variables related to gait, but with this technique, rodents are tested in an open field (OF). Thus, the (FPA/OF) testing enclosure is much less restrictive regarding the kinds of movements and ambulatory activity that may occur compared to motor responses exhibited in the narrow runways used with the DigiGait and CatWalk techniques. The FPA/OF allows for quantification of gait-related indices and movement patterns, as well as exploratory behaviors, which are classic open-field variables affected by alterations in emotionality and which may help further characterize the phenotype of the conditional UBC13 knockout mice.

In the present study, we evaluate motor coordination, gait, and movement patterns in conditional UBC13 knockout mice using three different testing methods: accelerating rotarod, DigiGait and FPA/OF. Use of the rotarod provides a standardized method for studying motor coordination, which is often used to assess compromised cerebellar functions. We have also tested the mice on the DigiGait system to determine how gait-related dependent variables change as a function of two different treadmill belt speeds (20 and 30 cm/s). Lastly, we have also utilized the FPA/OF technique to quantify gait-related variables and movement patterns during spontaneously occurring behaviors in an open-field environment.

## Methods

### Animals

Mice were purchased or maintained under pathogen-free conditions. All animal experimental protocols were approved by the Institutional Animal Care and Use Committees of Washington University School of Medicine and in accordance with the National Institute of Health guidelines. UBC13^*loxP/loxP*^ and *GABAα6-Cre* mice have been described^11,12^. The UBC13^*loxP/loxP*^ mice were backcrossed onto C57BL/6 background. For the conditional UBC13 knockout granule neuron specific mice (within the cerebellar cortex), *UBC13*^*loxP/loxP*^ mice were mated with transgenic mice expressing the recombinase Cre under the promoter of the *GABAα*6 gene^12^.

### Accelerating rotarod

Mice were first exposed to a 1-day habituation procedure on postnatal day 25 (P25), which included placing a mouse on a rod rotating at 5 rpm where it remained until it fell or a maximum of 60 s elapsed, and the time on the rod was recorded. This was repeated two more times (3 trials total) with no intertrial interval (ITI) intervening. If a mouse stayed on the rod for a total of 60 s over the three trials, then it qualified for testing on the accelerating rotarod trials on that day (baseline) and on the following 4 test days. The accelerating rotarod trial involved placing a mouse on the rod and then activating the program whereby the rotational speed of the rod increased from 0–40 rpm over 300s and the time spent on the rod was recorded. This sequence was repeated two more times with an ITI of 10 min for a total of three trials, which were conducted over five days when the mice were P26–P29 in age^4^. Averages of the 3 daily trials were used for statistical analyses.

### Gait dynamics

Gait dynamics were evaluated in the conditional UBC13 knockout (*UBC13*^*loxP/loxP*^; *GABAα6-Cre*) and littermate control (*UBC13*^*loxP/loxP*^) mice on postnatal days 27 or 28 (P27 or P28) using the DigiGait imaging system (Mouse Specifics Inc, Quincy, MA, USA) according to slightly modified procedures from those previously described^13,14^. Briefly, this technique involved taking digital images of the ventral surfaces of the paws of each mouse at 150 frames/sec as it ambulated on a moving transparent treadmill belt. Each mouse was tested individually on the treadmill, which was enclosed by a polycarbonate compartment (5 cm in width, 25 cm in length). Changes in the area of contact for each paw as it was being placed on the belt and as it was being lifted from it during a step were calculated and analyzed by system software, thus providing gait signals for each of the four limbs. Mice were given a habituation trial the day before actual testing was initiated. This involved placing a mouse in the apparatus and allowing it 3–5 minutes to explore the compartment while the treadmill remained stationary. After this brief acclimation period, movement of the treadmill belt was started at a speed of 10 cm/s, which was increased to 20 cm/s and then 30 cm/s. Mice were formally tested the next day when they were P27 or P28 of days of age. The mice were first evaluated at a belt speed of 20 cm/s, and if they successfully completed the trial in terms of yielding suitable data, then they were assessed at a belt speed of 30 cm/s. Although adult mice are typically observed to walk at a belt speed of 20 cm/s, the smaller body and the limbs of the juvenile mice resulted in them trotting at this belt speed. Approximately 5 s of video were collected from each mouse to provide an adequate number of sequential strides for quantification of several stride-related variables including swing and stance phases and braking and propulsion.

### Force Plate Actometer/Open-Field (FPA/OF)

Conditional UBC13 knockout and littermate control mice were individually evaluated on a FPA/OF test at P29–P31 to assess gait and other movement-related functions during spontaneously-occurring behaviors in an open field, according to previously-published methods^15,16^. A small subset of mice in each group were also assessed on the DigiGait procedure 2–3 days before being tested in the FPA/OF. Briefly, the apparatus is a square load plate (42 × 42 cm) that sits atop 4 load cell transducers (Honeywell/Sensotec, Columbus, OH) and is surrounded by 4 *Plexiglas* walls measuring 42.5 cm on each side and is 30.5 cm high. This size of the load plate was sufficiently large to allow mice to express their running behavior in uninterrupted sequences of locomotion suitable for gait analysis, but small enough to eliminate “galloping”. The 4 force transducers that supported the load plate at its corners were sampled 100 times/s (i.e., temporal resolution of 0.01 s). Force resolution was 0.2g-force, and spatial resolution was about 2 mm. A Visual Basic program written in house directed the timing and data-logging processes via a Measurement Computing USB 1208SF interface. A scrolling graphics program written in Visual Basic was used to extract selected gait metrics (e.g., velocity of runs, stride length and stride rate) from the data stream, and Pascal algorithms were used to extract the behavioral variables related to general activity and exploration of the field such as distance traveled. The recording (test) procedure involved placing a mouse in the center of the chamber and allowing it to explore for 20 min. Seven behavioral measures were quantified including gait-related variables such as stride length, stride rate, run velocity, and within-run-force-range, as well as general ambulatory/exploratory variables such as distance traveled, number of bouts of low mobility (tendency to restrict movement to small areas), and distance from the nearest wall during runs (thigmotaxis). All variables were analyzed as a function of test session half (i.e., first vs second) to detect changes over time. For gait analysis, each 20-min recording session was visualized with a scrolling graphics program that enabled the user to identify the distinctive rhythmic force-time wave forms (acquired at 100 samples/s) that accompany locomotion or “runs”. Also displayed were the spatial coordinates, x and y, of the lateral movements as a function of time. With a computer mouse-controlled cursor, the program user marked the beginning and ending of 20 separate runs that were 2.5 or more strides long. For each mouse, 10 runs were taken from the beginning of the recording session and 10 additional runs were taken from the end of the session and counting back toward the middle. The force-time information and corresponding spatial information were then subjected to a series of calculations that produced the gait metrics. For a run to qualify for inclusion in the analysis it had to have a nearly straight-line trajectory between its starting and ending points, no pausing, and be comprised of 2.5 or more strides. See Supplementary Methods for definition of terms and variables, and Supplementary Table 1 for an example of the derivation of values for variables from force-time waveforms.

### Statistical analyses

Repeated measures analysis of variance (rmANOVA) models containing one between-subjects variable (genotype) and one within-subjects (repeated measures) variable (trial, limb, or session half) were used to analyze the data. The Huynh-Feldt adjustment of alpha levels was utilized for all within-subjects effects containing more than two levels to protect against violations of sphericity/compound symmetry assumptions underlying rmANOVA models. Pairwise comparisons were conducted following appropriate significant over-all effects and evaluated against Bonferroni correction. Note that p < 0.00005 is presented when p = 0.0000.

## Results

Our recent identification of an RNF8/UBC13 ubiquitin signaling pathway controlling synapse formation and function in the cerebellum led us to further characterize this novel mechanism to enhance our current understanding of plasticity in cerebellar functions related to motor coordination^17–20^. For this purpose, we evaluated cerebellar-dependent motor performance of granule neuron-specific conditional UBC13 knockout mice on three different behavioral tests: accelerating rotarod; DigiGait, and FPA/OF.

### Conditional UBC13 knockout mice do not exhibit impaired performance on the accelerating rotarod

**Figure 1:**
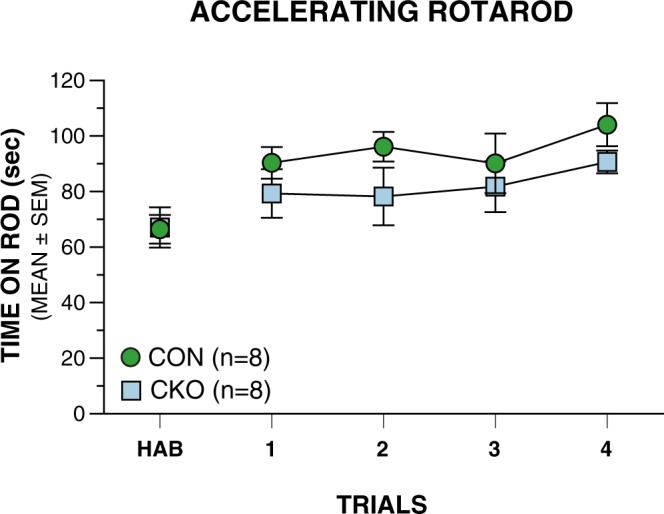
UBC13 conditional knockout (CKO) mice show a trend toward impairment on the accelerating rotarod relative to the littermate control (CON) group across 4 days of test trials  (genotype effect: *p* = 0.061) but these differences are not statistically significant.

A cohort of male conditional UBC13 knockout (n = 8) and male littermate control (n = 8) mice were evaluated on the accelerating rotarod, a standard test used to evaluate motor coordination in rodents with compromised cerebellar function. Analysis of performance during habituation showed that the conditional UBC13 knockout and control mice spent comparable amounts of time on the accelerating rotarod averaged across the trials [*t*(13) = 0.07, *p* = 0.95; Fig. 1). In addition, an rmANOVA indicated that, although there was a trend for the conditional UBC13 knockout mice to spend less time on the accelerating rotarod compared to the control group across 4 days of test trials, these differences were not statistically significant (*F*(1,14) = 4.14, *p* = 0.061).

### Conditional UBC13 knockout mice show robust deficits on gait-related variables during controlled speed, treadmill testing

In an effort to provide a more sensitive measure of motor function, we tested a larger cohort of conditional UBC13 knockout and littermate control mice, which included both males and females: conditional UBC13 knockout (n = 23; 9 M, 14 F); and littermate controls (n = 22; 14 M, 8 F). However, sex was not included as a between-subjects variable in the rmANOVAs conducted on the DigiGait data because numbers of males and females were unequal and disproportionately represented across genotypes, which could bias the effects involving this variable.

**Figure 2:**
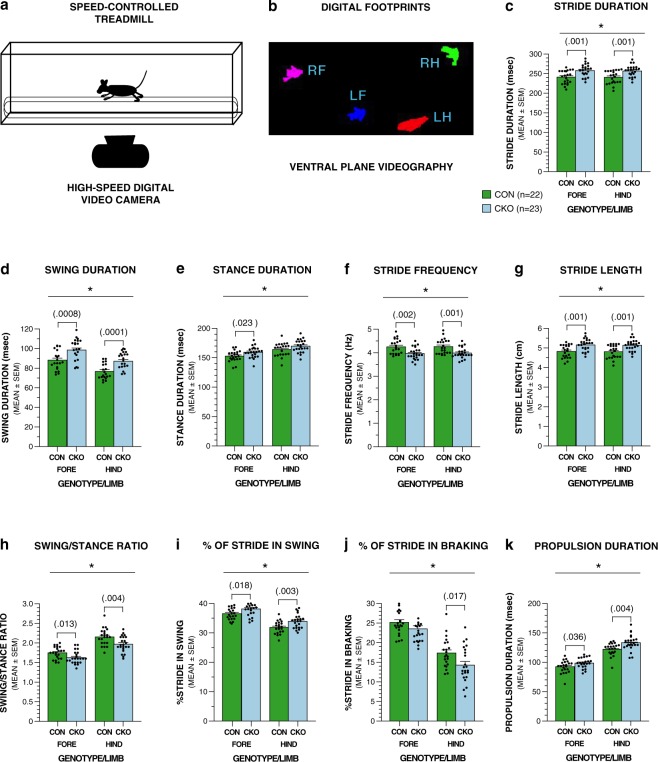
UBC13 CKO mice show significant performance deficits relative to littermate controls on gait-related variables when tested on the DigiGait procedure using a treadmill belt speed of 20 cm/s. (**a**) The DigiGait procedure involved having mice ambulate on a translucent treadmill (a) at controlled speeds (20 and 30 cm/s) on P27 or P28 to assess gait dynamics. (**b**) Ventral plane videography was used to acquire digital images of the ventral surfaces of the paws of each mouse at 150 frames/sec as it ambulated on the treadmill, with these images being used to derive several gait-related metrics. (**c**–**k**) Results from repeated measures (rm) ANOVAs conducted on the 20 cm/s treadmill speed data revealed significant (*) genotype effects signifying impaired performance in the UBC13 conditional knockout (CKO) mice compared to the littermate control (CON) group for (**c**) stride duration (*p = 0.001), (**d**) swing duration (*p = 0.0002), (**e**) stance duration (*p = 0.037), (**f**) stride frequency (*p = 0.001), (**g**) stride length (*p = 0.001), (**h**) swing-to-stance ratio (*p = 0.003), (**i**)% of stride in swing (*p = 0.003), (**j**) % of stride in braking (*p = 0.018), and (**k**) propulsion duration (*p = 0.004). Pair-wise comparisons were conducted following the finding of significant genotype effects and “*p*” values for the significant comparisons involving the forelimbs and/or hindlimbs are shown in brackets above the bars in the graphs. Dot plots representing the raw data are superimposed on the bar graphs.

Because gait dynamics vary as a function of speed during ambulation, the mice were evaluated at two different treadmill belt speeds (20 and 30 cm/s). Analysis of the data from the 20 cm/s trials showed that conditional UBC13 knockout mice were impaired relative to the control group on several classic stride-related variables with the swing phase appearing to be more greatly affected than the stance (Fig. 2). For example, a significant genotype effect was found for stride duration indicating that it was significantly increased in conditional UBC13 knockout mice relative to the littermate controls, which was seen in both forelimbs and hindlimbs (Fig. 2c; see Table 1 for rmANOVA results). A significant genotype effect was also found for swing duration, which was significantly increased for the conditional UBC13 knockout group in a similar manner for both limbs (Fig. 2d; Table 1). Stance duration was also observed to be increased in the conditional UBC13 knockout mice, but differences were only significant for the forelimbs (Fig. 2e; Table 1). Analyses of the stride frequency data revealed differences where levels were significantly decreased in the conditional UBC13 knockout group relative to the control mice in both limbs (Fig. 2f; Table 1), and consequently, stride length was significantly increased (Fig. 2g; Table 1) in an analogous manner in the mutant mice for both limbs. Further evidence for an alteration in the swing/stance phases of the conditional UBC13 knockout mice was provided by finding significant impairments in the swing-to-stance ratio, and percent of stride in swing variables (Fig. 2h–i; Table 1), with the greatest deficits observed in the hindlimbs. Significant genotype effects for percent of stride involved in braking, and propulsion duration suggested these components of the stance phase were abnormal in the conditional UBC13 knockout mice compared to the littermate controls with the greatest differences in the hindlimbs (Fig. 2j–k; Table 1).

**Table 1:** Means and ANOVA effects for treadmill gait variables (20 cm/s).

Variable (mean ± sd)	Overall/Limb Effects	F Statistics
Stride Duration (msec)	Genotype	*F*(1,43) = 12.40, *p* = 0.001
(CKO = 257.25 ± 14.74)	Forelimbs	*F*(1,43) = 11.93, *p* = 0.001
(CON = 240.83 ± 16.52)	Hindlimbs	*F*(1,43) = 12.21, *p* = 0.001
Swing Duration (msec)	Genotype	*F*(1,43) = 16.64, *p* = 0.0002
(CKO = 92.84 ± 8.24)	Forelimbs	*F*(1,43) = 12.87, *p* = 0.0008
(CON = 82.46 ± 8.84)	Hindlimbs	*F*(1,43) = 17.83, *p* = 0.0001
Stance Duration (msec)	Genotype	*F*(1,43) = 4.66, *p* = 0.037
(CKO = 164.54 ± 9.40)	Forelimbs	*F*(1,43) = 5.53, *p* = 0.023
(CON = 158.35 ± 9.83)	Hindlimbs	ns
Stride Frequency (Hz)	Genotype	*F*(1,43) = 11.99, *p* = 0.001
(CKO = 3.98 ± 0.23)	Forelimbs	*F*(1,43) = 11.52, *p* = 0.002
(CON = 4.26 ± 0.31)	Hindlimbs	*F*(1,43) = 11.80, *p* = 0.001
Stride Length (cm)	Genotype	*F*(1,43) = 12.59, *p* = 0.001
(CKO = 5.14 ± 0.29)	Forelimbs	*F*(1,43) = 11.90, *p* = 0.001
(CON = 4.81 ± 0.33)	Hindlimbs	*F*(1,43) = 12.58, *p* = 0.001
Swing-to-Stance Ratio	Genotype	*F*(1,43) = 10.28, *p* = 0.003
(CKO = 1.80 ± 0.15)	Forelimbs	*F*(1,43) = 6.77, *p* = 0.013
(CON = 1.95 ± 0.17)	Hindlimbs	*F*(1,43) = 9.19, *p = *0.004
% of Stride in Swing	Genotype	*F*(1,43) = 10.24, *p* = 0.003
(CKO = 36.03 ± 1.98)	Forelimbs	*F*(1,43) = 6.11, *p* = 0.018
(CON = 34.19 ± 1.89)	Hindlimbs	*F*(1,43) = 9.80, *p* = 0.003
% of Stride in Braking	Genotype	*F*(1,43) = 6.06, *p* = 0.018
(CKO = 18.90 ± 3.22)	Forelimbs	ns
(CON = 21.26 ± 3.22)	Hindlimbs	*F*(1,43) = 6.22, *p* = 0.017
Propulsion Duration (msec)	Genotype	*F*(1,43) = 9.55, *p* = 0.004
(CKO = 115.92 ± 8.93)	Forelimbs	*F*(1,43) = 4.69, *p* = 0.036
(CON = 107.32 ± 9.74)	Hindlimbs	*F*(1,43) = 9.38, *p* = 0.004

**Figure 3:**
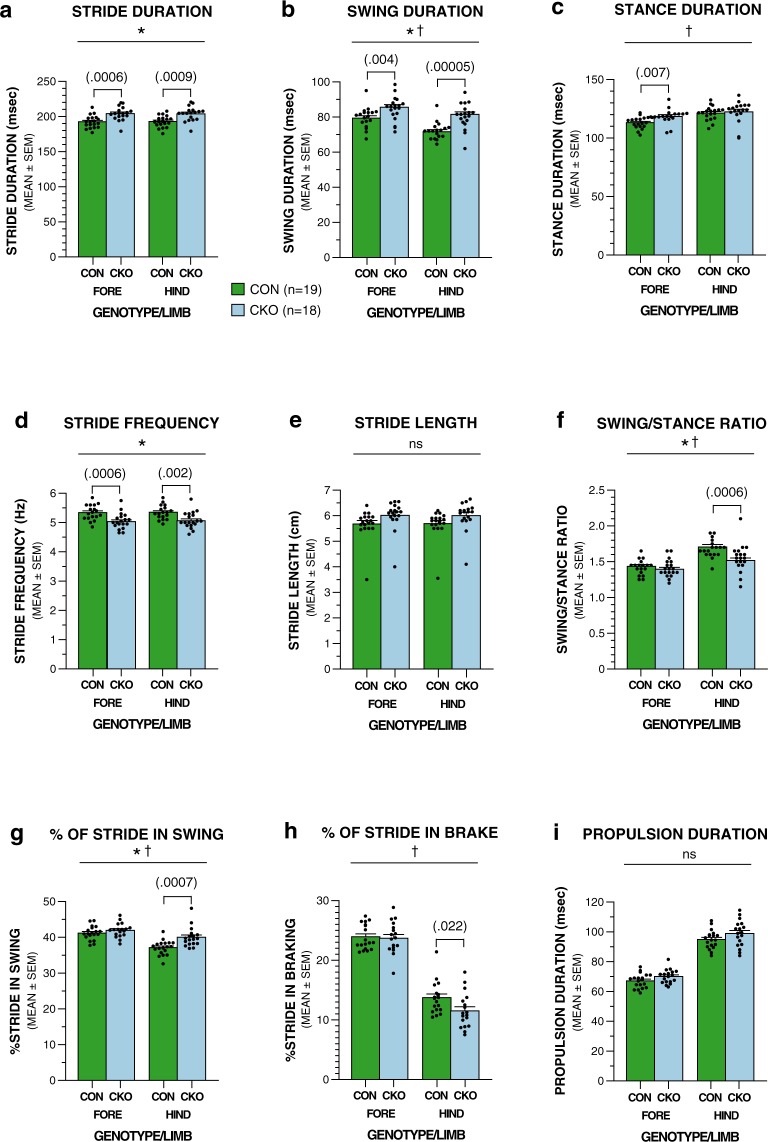
UBC13 CKO mice also exhibit deficits on several gait-related variables when tested on the DigiGait procedure using a higher treadmill belt speed (30 cm/s). If the UBC13 CKO and CON mice successfully completed tested on the DigiGait procedure using a belt speed of 20 cm/s, they were given additional trials at 30 cm/s. (**a**–**i**) Results from rmANOVAs conducted on the 30 cm/s treadmill speed data revealed significant genotype effects (*) or genotype x limb interactions (^†^) signifying impaired performance in the UBC13 CKO mice compared to the CON group for (**a**) stride duration (*p = 0.0006), (**b**) swing duration (*p = 0.0001; ^†^p = 0.006), (**c**) stance duration (^†^*p* = 0.008), (**d**) stride frequency (*p = 0.0009), (**f**) swing-to-stance ratio (*p = 0.006; ^†^*p* = 0.001), (**g**) % of stride in swing (*p = 0.007; ^†^*p* = 0.002), (**h**) % of stride in braking (^†^*p* = 0.039). Pair-wise comparisons were conducted following the finding of significant genotype effects and/or genotype x limb interactions, and “*p*” values for the significant comparisons involving the forelimbs and/or hindlimbs are shown in brackets above the bars in the graphs, and dot plots of the raw data are superimposed on the graphs. No significant effects involving genotype were found for stride length (**e**) or propulsion duration (**i**).

Several of the above findings were replicated when the mice were tested on the DigiGait procedure when the treadmill belt speed was increased to 30 cm/s, although sample sizes were slightly smaller since some mice from each group (5 conditional knockouts and 3 controls) were unable to run on the treadmill at this speed. Specifically, both stride and swing duration were found to be significantly increased in the conditional UBC13 knockout mice compared to the control group, and this was observed for the forelimbs and hindlimbs (Fig. 3a,b; Table 2). Also similar to the results from the 20 cm/s data set was the finding that stance duration was significantly increased for the conditional UBC13 knockout group only in the forelimbs (Fig. 3c; Table 2). Analysis of stride frequency indicated again that levels were significantly decreased in the conditional UBC13 knockout mice compared to the control group for both forelimbs and hindlimbs (Fig. 3d; Table 2). However, in contrast to the findings from the lower belt speed, analysis of stride length did not yield a significant genotype effect (Fig. 3e, Table 2) for the 30 cm/s condition. This appears to be due to one outlier in each group. Analysis of the swing-to-stance ratio and the percent of stride in swing revealed significant genotype and genotype x limb interaction effects with subsequent pair-wise comparisons showing that these effects were due to significant deficits on the part of the conditional UBC13 knockout mice only in the hindlimbs (Fig. 3f–g; Table 2). The percent of stride involved in braking was significantly reduced in the conditional UBC13 knockout group relative to the control mice, but also only in the hindlimbs (Fig. 3h; Table 2). Lastly, similar to the stride length results, analysis of the propulsion duration data at the higher belt speed did not produce a significant genotype effect (Fig. 3i, Table 2). These results suggest that conditional knockout of UBC13 is associated with discoordinated gait where regular, fluid striding, may be disrupted by alterations in stride frequency and length, resulting from modified stance and swing phases. A decrease in the percent of stride involved in braking, particularly at the higher belt speed, also likely contributed to the abnormal gait in the conditional UBC13 knockout mice.

**Table 2:** Means and ANOVA effects for treadmill gait variables (30 cm/s).

Variable (mean ± sd)	Overall/Limb Effects	F Statistics
Stride Duration (msec)	Genotype	*F*(1,35) = 14.04, *p* = 0.0006
(CKO: 203.88 ± 9.84)	Forelimb	*F*(1,35) = 14.44, *p* = 0.0006
(CON: 192.57 ± 8.50)	Hindlimb	*F*(1,35) = 13.24, *p* = 0.0009
Swing Duration (msec)	Genotype	*F*(1,35) = 19.72, *p* = 0.0001
(CKO: 83.51 ± 5.55)	Genotype × Limb	*F*(1,35) = 8.75, *p* = 0.006
(CON: 75.45 ± 5.50)	Forelimb	*F*(1,35) = 9.48, *p* = 0.004
	Hindlimb	*F*(1,35) = 29.69, *p* < 0.00005
Stance Duration (msec)	Genotype	ns
(CKO: 120.36 ± 7.48)	Genotype × Limb	*F*(1,35) = 8.00, *p* = 0.008
(CON: 117.16 ± 5.12)	Forelimb	*F*(1,35) = 8.08, *p* = 0.007
	Hindlimb	ns
Stride Frequency (Hz)	Genotype	*F*(1,35) = 13.08, *p* = 0.0009
(CKO: 5.05 ± 0.27)	Forelimb	*F*(1,35) = 14.07, *p* = 0.0006
(CON: 5.35 ± 0.24)	Hindlimb	*F*(1,35) = 11.72, *p* = 0.002
Stride Length (cm)	Genotype	ns
(CKO: 6.01 ± 0.58)	Genotype × Limb	ns
(CON: 5.68 ± 0.58)		
Swing-to-Stance Ratio	Genotype	*F*(1,35) = 8.69, *p* = 0.006
(CKO: 1.46 ± 0.11)	Genotype × Limb	*F*(1,35) = 12.85, *p* = 0.001
(CON: 1.57 ± 0.12)	Forelimb	ns
	Hindlimb	*F*(1,35) = 14.30, *p* = 0.0006
% of Stride in Swing	Genotype	*F*(1,35) = 8.33, *p* = 0.007
(CKO: 40.98 ± 2.11)	Genotype × Limb	*F*(1,35) = 10.98, *p* = 0.002
(CON: 39.14 ± 1.76)	Forelimb	ns
	Hindlimb	*F*(1,35) = 13.87, *p* = 0.0007
% of Stride in Braking	Genotype	ns
(CKO: 17.60 ± 2.43)	Genotype × Limb	*F*(1,35) = 4.58, *p* = 0.039
(CON: 18.83 ± 1.95)	Forelimb	ns
	Hindlimb	*F*(1,35) = 5.76, *p* = 0.022
Propulsion Duration (msec)	Genotype	ns
(CKO: 84.44 ± 5.92)	Forelimb	ns
(CON: 80.99 ± 4.92)	Hindlimb	ns

### Alteration of spontaneously-occurring motor behaviors in conditional UBC13 knockout mice tested in an open field

As noted above, evaluating the performance of the mice with the DigiGait procedure revealed a profile of deficits on several gait indices in the conditional UBC13 knockout group at two different treadmill speeds. Because several aspects of ambulation such as speed and available space are highly restricted using this technique, we also assessed performance of the mice on the FPA/OF test and quantified gait and general locomotor/exploratory variables during spontaneously-occurring motor behavior in an open-field environment. Behavioral variables collected during FPA/OF testing were analyzed in conditional UBC13 knockout (n = 20; 11 M, 9 F), and littermate control mice (n = 18; 5 M, 13 F) as a function of time in the test session (first half vs second half) they were measured, as several aspects of locomotor/exploratory behaviors change over time under such conditions. The sex variable was also not formally evaluated in the FPA/OF data set because of the possibility that unbalanced numbers of males and females across genotypes might produce unwanted biases regarding sex-related effects.

**Figure 4:**
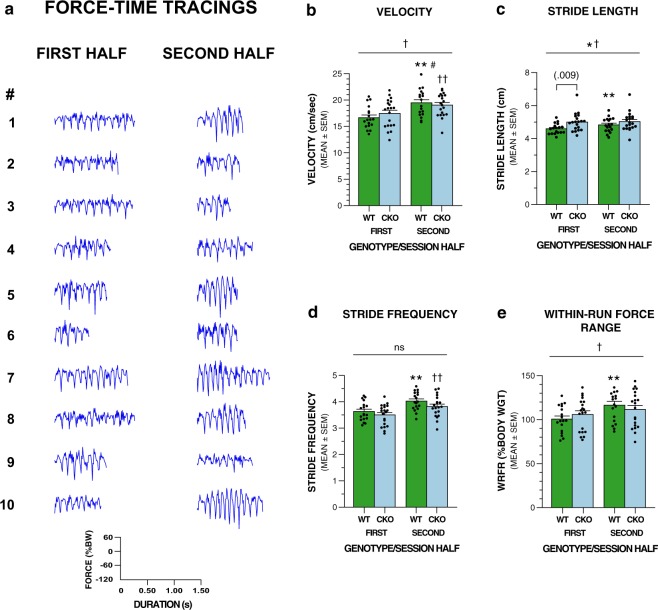
UBC13 CKO mice display impaired performance on gait-related measures during spontaneously-occurring ambulation compared to littermate controls when tested on the force-plate actometer/open field apparatus (FPA/OF) on P29-P31 for one, 20-min session. (**a**) Force-time waveforms from a representative control mouse across 10 bouts (^#^) of trotting in the first and 10 in the second halves of the testing session are shown illustrating the kind of information used to extract certain gait-related parameters from continuously-recorded ground reaction forces. Note the general changes in the waveforms between bouts of trotting during the first and second session halves. See Supplementary Table 1 showing values for variables extracted from these force-time waveforms. (**b**–**e**) Analysis of data extracted from the force-time profiles from each mouse were used to quantify several gait-related variables including velocity, stride length, stride frequency, and within-run force range. (**b**) A significant genotype x session half interaction was found for movement velocity (^†^p = 0.030), but both the UBC13 CKO and CON littermates each significantly increased their velocities across session halves (**p = 0.0002 and ^††^*p* < 0.00005, respectively), although the CON group showed a greater percent increase (^#^p = 0.046). (**c**) A significant genotype effect (*p < 0.05) and genotype x session half interaction (^†^*p* = 0.003) were found for stride length, which appears mostly due to UBC13 CKO mice having significantly longer stride lengths than the CON group for the first session half. Also, the stride lengths of the CON mice were greater in the second session half (**p = 0.0005), while those of the UBC13 CKO mice were not. (**d**) Both the UBC13 CKO and CON groups exhibited significant increases in stride frequency in the second session half relative to the first half although no significant effects involving genotype were observed (**p < 0.00005 and ^††^*p* < 0.00005, respectively). (**e**) A significant genotype x session half interaction (^†^*p* < 0.00005) was found for the within-run force range data, which appears to be mostly due to the CON group showing significantly increased levels during the second half of the test session (**p < 0.00005) while those of the UBC13 CKO mice were unchanged across session halves, and no group differences were observed for either half session.

Four gait-related variables were quantified during the FPA/OF test: velocity; stride length; stride frequency and within-run force range. Velocities measured during spontaneous straight runs within the field were somewhat less than the 20 cm/s rate that was imposed on the mice during DigiGait testing at the lower belt speed, and rates varied across test session halves. Specifically, movement velocities were observed to be roughly similar in the two groups for each test session half, averaging around 16.5 to 17.5 cm/s for the first half, although the conditional UBC13 knockout and littermate control groups each significantly increased their ambulation velocities in the second half of the test session (19.0 to 19.5 cm/s, respectively) relative to the first half (Fig. 4b, Table 3). However, an rmANOVA conducted on these data yielded a significant genotype x session half interaction suggesting that the increases in velocities across halves were not equivalent for each group (Table 3). Additional analyses confirmed that the control mice showed a significantly greater percent increase in velocity across session halves compared to the conditional UBC13 knockout mice, [*F*(1,36) = 4.29, *p* = 0.046]. Stride length was also quantified during the FPA/OF test. An rmANOVA conducted on these data revealed a significant genotype effect and genotype x session half interaction (Fig. 4c, Table 3), thus confirming the earlier DigiGait results showing that stride was significantly lengthened in the conditional UBC13 knockout mice relative to littermate controls at 20 cm/s. The genotype x session half interaction generally reflected that the conditional UBC13 knockout mice had approximately equivalent stride lengths across first and second session halves, while the littermate controls significantly increased their stride lengths in the second half of the session. Subsequent pair-wise comparisons showed that the conditional UBC13 knockout mice exhibited significantly increased stride lengths relative to the control group for the first half of the test session but not during the second session half (Fig. 4c, Table 3). Another gait-related variable, stride frequency, was significantly increased in the second half compared to the first half session in both groups, but the genotype and genotype x session half interaction effects were not significant (Fig. 4d, Table 3). This finding is in contrast to the stride frequency results derived during DigiGait testing when stride frequency was found to be significantly reduced in the conditional UBC13 knockout mice compared to the control group. The within-run force range (WRFR), a metric reflecting the regularity of rhythmicity of force variation during ambulation, was also analyzed but did not yield a significant main effect of genotype, although a significant genotype x session half interaction was found (Fig. 4e, Table 3). The interaction effect appeared mostly due to the control mice showing a significant increase in WRFR during the second half of the session compared to the first, while the conditional UBC13 knockout group did not show a significant change across the two session halves. The WRFR results raise the possibility that the conditional UBC13 knockout mice are less likely to vary their force profiles during runs affected by changes in behavioral reactivity during habituation to the novel test environment.

**Table 3:** Means and ANOVA effects for force plate actometer/open field variables.

Variable (mean ± sd)	Overall Effects	F Statistics
**Gait**		
Velocity (cm/s)	Genotype (Geno)	ns
(CKO: 18.26 ± 2.31)	Session Half (SH)	*F*(1,36) = 62.59, *p* < 0.00005
(CON: 18.07 ± 2.13)	Geno × SH	*F*(1,36) = 5.12, *p* = 0.030
	SH1 (between groups)	ns
	SH2 (between groups)	ns
	CKO: SH1 vs SH2	*F*(1,36) = 16.84, *p* = 0.0002
	CON: SH1 vs SH2	*F*(1,36) = 49.17, *p* < 0.00005
Stride Length (cm)	Genotype	*F*(1,36) = 4.11, *p* < 0.05
(CKO: 5.01 ± 0.54)	Session Half (SH)	*F*(1,36) = 9.91, *p* = 0.003
(CON: 4.71 ± 0.34)	Geno × SH	*F*(1,36) = 5.83, *p* = 0.021
	SH1 (between groups)	*F*(1,36) = 7.63, *p* = 0.009
	SH2 (between groups)	ns
	CKO: SH1 vs SH2	ns
	CON: SH1 vs SH2	*F*(1,36) = 14.69, *p* = 0.0005
Stride Frequency (Hz)	Genotype	ns
(CKO: 3.66 ± 0.37)	Session Half (SH)	*F*(1,36) = 51.17, *p* < 0.00005
(CON: 3.83 ± 0.32)	Geno × SH	ns
	SH1 (between groups)	ns
	SH2 (between groups)	ns
	CKO: SH1 vs SH2	*F*(1,36) = 21.47, *p* < 0.00005
	CON: SH1 vs SH2	*F*(1,36) = 29.85, *p* < 0.00005
Within-run Force Range (%bw)	Genotype	ns
(CKO: 108.72 ± 18.58)	Session Half (SH)	*F*(1,36) = 23.01, *p* < 0.00005
(CON: 108.49 ± 15.30)	Geno × SH	*F*(1,36) = 5.07, *p* = 0.031
	SH1 (between groups)	ns
	SH2 (between groups)	ns
	CKO: SH1 vs SH2	ns
	CON: SH1 vs SH2	*F*(1,36) = 23.60, *p* < 0.00005
**Open Field**		
Distance (cm)	Genotype (Geno)	ns
(CKO: 2155.81 ± 453.46)	Session Half (SH)	*F*(1,36) = 101.85, *p* < 0.00005
(CON: 2222.69 ± 385.36)	Geno × SH	ns
	SH1 (between groups)	ns
	SH2 (between groups)	ns
	CKO: SH1 vs SH2	*F*(1,36) = 61.65, *p* < 0.00005
	CON: SH1 vs SH2	*F*(1,36) = 41.76, *p* < 0.00005
Low Mobility Bouts	Genotype	ns
(CKO: 22.00 ± 9.58)	Session Half (SH)	*F*(1,36) = 75.54, *p* < 0.00005
(CON: 18.86 ± 8.82)	Geno × SH	ns
	SH1 (between groups)	ns
	SH2 (between groups)	ns
	CKO: SH1 vs SH2	*F*(1,36) = 51.98, p < 0.00005
	CON: SH1 vs SH2	*F*(1,36) = 26.43, p < 0.00005
Distance from Wall (cm; Thigmotaxis)	Genotype	ns
(CKO: 6.75 ± 1.73)	Session Half (SH)	*F*(1,36) = 65.94, *p* < 0.00005
(CON: 6.60 ± 1.36)	Geno × SH	*F*(1,36) = 5.49, *p* = 0.025
	SH1 (between groups)	ns
	SH2 (between groups)	ns
	CKO: SH1 vs SH2	*F*(1,36) = 17.61, *p* = 0.0002
	CON: SH1 vs SH2	*F*(1,36) = 52.01, *p* < 0.00005

**Figure 5:**
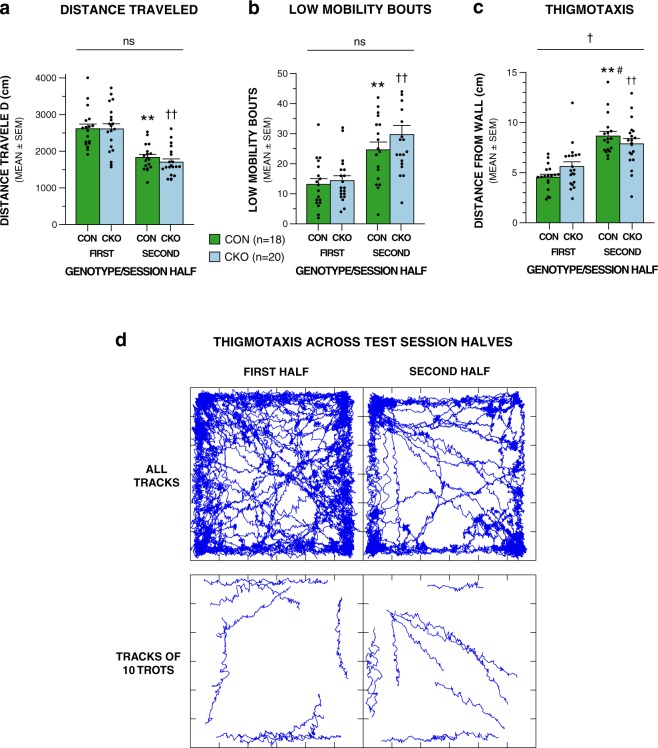
“Time on task” (test session half) effects on open-field behaviors suggest possible alterations in emotionality in UBC13 CKO mice. No significant effects involving genotype were found for either distance traveled (**a**) or low mobility bouts (**b**), although the UBC13 CKO and CON groups each showed significant decreases in distance traveled during the second test session half versus the first, with corresponding increases observed in low mobility bouts for the second session half versus the first (**p < 0.00005 and ^††^*p* < 0.00005, respectively for each variable). (**c**) However, a significant genotype x session half interaction (^†^*p* < 0.00005), was observed for the thigmotaxis data, being mostly due to the CON mice staying closer to the nearest wall during the first session half, but farther away in the second session half compared to the UBC13 CKO group. Nevertheless, a general reduction in thigmotaxis across session halves was observed for both the UBC13 CKO and CON groups, each showing significantly decreased distances away from the nearest wall during the second session half relative to the first (**p = 0.0002, ^††^*p* < 0.00005, respectively). Importantly, a significantly greater percentage change in thigmotaxis was exhibited by the CON mice compared to the UBC13 CKO group (^#^*p* = 0.015) across session halves, suggesting that the two groups differed in their emotional response in habituating to the test environment, with the UBC13 CKO mice being less responsive. (**d**) Run trajectories (ambulatory paths) from a control mouse are shown in the top row. These trajectories represent all tracks recorded (all movements in the x-y plane) during the two session halves and depict overall differences between the halves, and the proclivity of the mouse to remain near the chamber wall during the first half, which decreases in the second. Trajectories in the bottom row are from the first 10 tracks (left) and last 10 tracks (right) of movements that qualify as trots (runs) showing thigmotaxis being exhibited while the mouse was locomoting, thus demonstrating that low mobility bouts (sleeping or staying in one place near the wall) did not totally account for avoidance of the center of the field (first half), which decreases across session halves.

Three standard variables related to locomotion and exploration were also quantified during the FPA/OF test, and both groups showed typical changes for some of these across session halves. For example, distance traveled was higher in the first half of the test session versus the second, while the number of low mobility bouts increased in the second half relative to the first (Fig. 5a,b; Table 3). Neither of these two variables was affected by genotype, nor were any interactions between genotype and session half detected. Distance from the nearest wall during runs, considered to be an index of altered emotionality exhibited by rodents in an open field, was also quantified. Analysis of these data showed that distance from the nearest wall significantly increased from the first to the second half of the session for both groups (Fig. 5c; Table 3), indicating that thigmotaxis (remaining in the periphery of the field near the walls), lessened as the mice became more familiar with the test environment. Although no main effect of genotype was observed, there was a significant genotype x session half interaction (Table 3). These data show that the interaction was the result of conditional UBC13 knockout mice having higher values (greater distance from the wall) than the control mice in the first half of the test session and lower values than the control group in the second half. Importantly, although both groups exhibited large increases in distance from the wall in the second half of the session compared to the first half, the control mice showed a significantly greater percentage change compared to the conditional UBC13 knockout group [*F*(1,36) = 6.51, *p* = 0.015]. These results suggest that the two groups exhibited differing degrees of thigmotaxis in adapting (habituating) to the environmental conditions, with the conditional UBC13 knockout mice somewhat less reactive compared to littermate controls.

## Discussion

In this study, we extend our characterization of the functional impairments accruing from conditional knockout of the ubiquitin-conjugating E2 enzyme UBC13 in granule neurons of the mouse cerebellum. Conditional knockout of UBC13 significantly increased the number of parallel fiber presynaptic boutons and functional parallel fiber/Purkinje cell synapses, and these alterations in neurodevelopment are associated with deficits in cerebellar-dependent learning^4^. Here, we show that conditional UBC13 knockout mice exhibit reliable deficits on several gait-related variables when their velocity of movement and available space for locomotion is highly restricted. In addition, we also find that selected gait parameters and those related to spontaneous exploration in an open field may be affected in conditional UBC13 knockout mice. These findings advance our understanding of how impairment of granule neuron connectivity in the developing brain may affect subsequent motor performance and learning, and why such dysfunctions may be present in neurodevelopmental disorders such as ASD.

Analysis of the accelerating rotarod data did not reveal impaired performance in the conditional UBC13 knockout mice, although they exhibited a trend towards a deficit relative to littermate controls (genotype effect: *p* = 0.06). Notably, however, while the accelerating rotarod is a standard test used to assess cerebellar dysfunction, its use may not reveal deficits following developmental insult to cerebellar granule cells such as those resulting from neonatal dexamethasone exposure^21^ or genetic mutation involving deficiency of the nuclear orphan receptor TAK1^22^. Considering this, we proceeded to evaluate a larger cohort of mice with the DigiGait procedure, which reduces variability by controlling ambulatory speed and available space for locomotion. The conditional UBC13 knockout mice were impaired on several common gait-related variables, including duration of stride, swing, stance, and propulsion, as well as stride frequency, stride length, swing/stance ratio, percent of stride in swing, and percent of stride in brake when tested on the DigiGait procedure at a treadmill belt speed of 20 cm/s. Robust genotype effects (p < 0.005) were found for the majority of gait metrics that were evaluated using the DigiGait procedure and most of these were replicated across two treadmill speeds. These results provide strong evidence that conditional UBC13 knockout mice have an abnormal gait, likely resulting from alterations in the stance and swing phases, suggesting that UBC13 ubiquitin signaling in granule neurons participates in cerebellar programs related to certain coordinated movements.

Some of our DigiGait findings in conditional UBC13 knockout mice are consistent with those reported for a mouse model of ASD that included impaired motor control and learning resulting from deficits in cerebellar plasticity^20^. Impairments in this mouse model (patDp/+) for the human 15q11-13 duplication^20^, one of the most frequently observed genetic aberrations in ASD^23^, also featured longer stride lengths, reduced stride frequency, and enhanced propulsion duration as quantified by the DigiGait procedure. The patDp/+model mice do not exhibit impaired rotarod performance, although these mice have deficits in delayed eye blink conditioning similar to the eye blink conditioning deficits in conditional UBC13 knockout mice^4^. In addition, mice with a null mutation of cerebellin1, a granule neuron glycoprotein essential for synapse formation^24,25^, have hindlimb movement impairments as assessed by kinematic analyses of ambulation^26^. Moreover, injecting recombinant Cbln1 protein into the cerebellum restored synapse formation between parallel fiber/Purkinje cells and improved aspects of hindlimb function^25,26^ suggesting that dysfunction of parallel fiber/Purkinje cell synapses might underlie the movement disturbances. There are several design features of our and the Takeuchi *et al*., studies that make it difficult to compare results, including differing gait analyses, ages, treadmill speeds, and the fact that UBC13 was knocked out only and specifically in granule neurons. However, taken together, these studies suggest that either large increases or decreases in parallel fiber/Purkinje cell synapse numbers may lead to demonstrable gait impairments.

As noted above, various indices related to gait and locomotion may be affected by several influences such as velocity of movement, whether locomotion is forced or spontaneous, the amount of space in which ambulation is measured, and psychological factors. Results from the FPA/OF test demonstrated that the spontaneously-occurring movement velocities in the mice were slightly less than the 20 cm/s belt speed used for one of the DigiGait trials. Importantly, there was a high concordance of stride length values averaged across test session halves from the FPA/OF procedure versus those generated by the DigiGait procedure, varying less than 0.1 cm for either group across the two tests. Moreover, stride length was found to be significantly reduced in the conditional UBC13 knockout group compared to the control mice when the data were analyzed from both the FPA/OF and DigiGait (20 cm/s) procedures. Other notable OF/FPA test results also included significant increases in both stride length and stride frequency during the second test session half compared to the first half in both groups. However, in contrast to the stride length results, analysis of the stride frequency FPA/OF data did not reveal differences between groups, whereas differences had been observed on this variable using the DigiGait procedure. There was a greater disparity (4-fold) in the stride frequency values across the two techniques compared to those observed for stride length, and there was substantially greater variability in stride frequency values when quantified using the FPA/OF technique compared to those derived from the DigiGait procedure, which may account for the lack of differences between groups on the former measure. This increased variability may reflect the lack of constraints on ambulation during the FPA/OF compared to the DigiGait procedure, and also an effect of “time on task” was not likely to be an influence during the DigiGait testing since the test trial was so brief. Test session half was also an important variable with regard to the velocity and WRFR data from the FPA/OF measure. For example, the control mice exhibited a greater percent increase in velocity across session halves compared to the conditional UBC13 knockout mice. Furthermore, the control mice also showed a significant increase in WRFR levels during the second half of the session compared to the first, while conditional UBC13 knockout mice did not. These results suggest that some components of gait, like movement velocity and force variation during runs, are less likely to be affected by psychological factors in the conditional UBC13 knockout mice than in littermate controls, such as those associated with adaptation/habituation to a novel environment.

Analysis of the standard open-field variables related to locomotion and exploration indicated that the conditional UBC13 knockout and control mice displayed similar levels of ambulatory activity in terms of distance traveled and with regard to times spent at rest as indexed by low mobility bouts. Moreover, each group exhibited comparable effects of test session half by traveling shorter distances in the first half relative to the second half, while low mobility bouts were observed to increase in the second half compared to the first half. In contrast, analysis of the thigmotaxis (distance from the nearest wall) data provided some evidence that session half had less of an effect on the conditional UBC13 knockout group than on littermate controls, since the latter showed a significantly greater percent change across session halves. These findings suggest that the two groups exhibited differing patterns of emotionality in adapting to the environmental conditions, with the conditional UBC13 knockout mice somewhat less reactive across the session halves compared to the control mice.

Other results from the present study suggest that psychological factors may influence gait, in general, when quantified in an open field. For example, the finding that velocity was significantly higher in the second half compared to the first half of the session for both groups was unexpected given the decrease in distance traveled during the second half relative to the first for both groups. To our knowledge this result has not been reported previously, which may reflect that our methodology underpinning this observation has not heretofore been used for this purpose. Essentially, this result suggests a partial dissociation between distance traveled and some parameters of individual discrete episodes of running in the open field. A somewhat similar interpretation has been offered^27^ concerning how levels of emotionality may affect stride length and stride rate, although a different experimental design and test methodologies were used compared to those in the present study. Instead of interpreting data from within-session effects during testing in an open field as we have done, these authors drew conclusions from mouse strain comparisons (anxious versus less anxious) and from environmental manipulations designed to alter aspects of emotionality. These authors interpreted their data as providing evidence that mice alter their gait, as well as their posture and position in space to stabilize their locomotion in potentially threatening environments, but also modify some of aspects of locomotion to enhance propulsion and maneuverability in environs perceived to be less threatening.

Cerebellar dysfunction has been associated with alterations in emotionality as a result of findings in human patients with neurologic disease confined to the cerebellum, which has been labeled “cerebellar cognitive affective syndrome”^28^. A subset of patients in this study exhibited personality changes including passivity, blunting of affect, and disinhibited behavioral responses. In addition, genes encoding cbln1 and its receptor have been reported to be associated with several psychiatric disorders, but it is unclear whether and what kinds of cognitive impairments accrue from disturbances solely in cerebellar function^29^. Results from studies conducted in animal models involving *cbln1*-null mice suggest that dysfunction in parallel fiber/Purkinje cell synapses may affect cognitive capabilities and alter emotionality^30^. In particular, these authors showed that Cerebellin 1 signaling mediates specific aspects of fear conditioning and spatial memory, indicating that Cbln1 signaling influences cognitive as well as motor functions. For example, these authors reported that *cbln1* null mice and mice that were deficient of Cbln1 primarily in the cerebellum (*cb-cbln1* null mice), each remained in the central area of an open-field for a greater period of time during a brief (5-min) test compared to controls, suggesting less anxiety-like behavior, and each also exhibited acquisition deficits in contextual fear and auditory cue conditioning. These findings raise the possibility that the disrupted gait in conditional UBC13 knockout mice may accrue from not only motor disturbances but also from altered emotionality and other psychological factors, and that this may have relevance for human patients with cerebellar dysfunction.

In summary, our results show that abnormal neurodevelopment of cerebellar granule cell circuits resulting from conditional knockout of UBC13 is associated with significant impairment in several gait-related indices when measured under highly-controlled conditions, as well as with alteration in some aspects of spontaneously-occurring locomotion. Preliminary evidence from our FPA/OF test results suggest that the emotional response of conditional UBC13 knockout mice to varying environmental conditions (e.g., habituation to novelty) may be different from control mice and that this may affect gait and general locomotor behavior. The conditional UBC13 knockout mouse is valuable for not only studying the effects of disturbances in cerebellar granule cell circuitry on gait and other aspects of locomotion, but given the observed motor deficits and possible alteration of emotionality in these mutant mice, it may provide an informative model for studying analogous behavioral anomalies in neurodevelopmental disorders resulting from deregulation of ubiquitin signaling such as occurs in ASD and Angelman syndrome. Other in-depth studies are required, however, to determine whether the nature of the altered emotionality in conditional UBC13 knockout mice is analogous to those observed in ASD or other neurodevelopmental disorders, and whether additional aspects of the behavioral phenotype are representative.

## Supplementary information

Supplementary Information

## Data Availability

The datasets generated during and/or analyzed during the current study are available from the corresponding author upon reasonable request.

## References

[1] Glessner JT (2009). Autism genome-wide copy number variation reveals ubiquitin and neuronal genes. Nature.

[2] Lehman NL (2009). The ubiquitin proteasome system in neuropathology. Acta Neuropathol..

[3] Kishino T, Lalande M, Wagstaff J (1997). UBE3A/E6-AP mutations cause Angelman syndrome. Nat. Genet..

[4] Valnegri P (2017). RNF8/UBC13 ubiquitin signaling suppresses synapse formation in the mammalian brain. Nat. Commun..

[5] Altman, J. & Bayer, S. A. *Development of the cerebellar system: in relation to its evolution*, *structure*, *and functions*. pp. 783 (CRC Press, Boca Raton, 1997).

[6] Ghez, G. & Thach, W. T. In *P*rinci*p*les o*f neural science*, *Kandel*, E. R., Schwartz, J. H., Jessell, T. M., Eds, chap. 42, pp. 832–952 (McGraw-Hill, Health Professions Division, New York, 2000).

[7] Heglund NC, Taylor CR, McMahon TA (1974). Scaling stride frequency and gait to animal size: mice to horses. Science.

[8] Hruska RE, Kennedy S, Silbergeld EK (1979). Quantitative aspects of normal locomotion in rats. Life Sci..

[9] Neckel ND (2015). Methods to Quantify the Velocity Dependence of Common Gait Measurements from Automated Rodent Gait Analysis Devices. J. Neurosci. Methods.

[10] Batka RJ (2014). The Need for Speed in Rodent Locomotion Analyses. Anat. Rec. (Hoboken).

[11] Yamamoto M (2006). Key function for the Ubc13 E2 ubiquitin-conjugating enzyme in immune receptor signaling. Nat. Immunol..

[12] Fünfschilling U, Reichardt LF (2002). Cre-mediated recombination in rhombic lip derivatives. Genesis.

[13] Puram SV (2011). A TRPC5-regulated calcium signaling pathway controls dendrite patterning in the mammalian brain. Genes Dev..

[14] Suidan, G. L. *et al*. Lack of Tryptophan Hydroxylase-1 in Mice Results in Gait Abnormalities. *PLoS One* **8**, e59032, www.plosone.org (2013) 10.1371/journal.pone.0059032 PMC3597584 23516593

[15] Dearborn JT (2015). Comprehensive functional characterization of murine infantile Batten disease including Parkinson-like behavior and dopaminergic markers. Sci. Rep..

[16] Fowler SC, Moshera LJ, Godarb SC, Bortolato M (2017). Assessment of gait and sensorimotor deficits in the D1CT-7 mouse model of Tourette syndrome. J. Neurosci. Methods.

[17] De Zeeuw CI, Ten Brinke MM (2015). Motor learning and the cerebellum. Cold Spring Harb. Perspect. Biol..

[18] Gao Z, van Beugen BJ, De Zeeuw CL (2012). Distributed synergistic plasticity and cerebellar learning. Nature Rev. Neurosci..

[19] Heiney SA, Wohl MP, Chettih SN, Ruffolo LI, Medina JF (2014). Cerebellar-dependent expression of motor learning during eyeblink conditioning in head-fixed mice. J. Neurosci..

[20] Piochon C (2014). Cerebellar Plasticity and Motor Learning Deficits in a Copy Number Variation Mouse Model of Autism. Nat. Commun..

[21] Maloney SE (2011). Long-term effects of multiple glucocorticoid exposures in neonatal mice. Behav. Sci..

[22] Kim YS (2010). Altered cerebellar development in nuclear receptor TAK1/TR4 null mice is associated with deficits in GLAST(+) glia, alterations in social behavior, motor learning, startle reactivity, and microglia. Cerebellum.

[23] Nakatani J (2009). Abnormal behavior in a chromosome-engineered mouse model for human 15q11-13 duplication seen in autism. Cell..

[24] Hirai H (2005). Cbln1 is essential for synaptic integrity and plasticity in the cerebellum. Nat. Neurosci..

[25] Ito-Ishita A (2008). Cbln1 regulates rapid formation and maintenance of excitatory synapses in mature cerebellar Purkinje cells *in vitro* and *in vivo*. J. Neurosci..

[26] Takeuchi E, Ito-Ishida A, Michisuke Yuzaki M, Yanagihara D (2018). Improvement of cerebellar ataxic gait by injecting Cbln1 into the cerebellum of *cbln1*-null mice. Sci. Rep..

[27] Lepicard EM (2006). Spatio-temporal analysis of locomotion in BALB/cByJ and C57BL/6J mice in different environmental conditions. Behav. Brain Res..

[28] Schmahmann JD, Sherman JC (1998). The cerebellar cognitive affective syndrome. Brain.

[29] Hampson DR, Blatt GJ (2015). Autism spectrum disorders and neuropathology of the cerebellum. Front. Neurosci..

[30] Otsuka S (2016). Roles of Cbln1 in Non-Motor Functions of Mice. J. Neurosci..

