# Supplementary material for: Conditional knockout of UBC13 produces disturbances in gait and spontaneous locomotion and exploration in mice
Source: Sci Rep. 2019 Mar 13;9:4379. doi: 10.1038/s41598-019-40714-3 (PMC6416404; doi:10.1038/s41598-019-40714-3)
Supplement: Supplementary file 1 — Supplementary Information [file 41598_2019_40714_MOESM1_ESM.pdf]

Supplementary Information (3 pages)

Conditional knockout of UBC13 produces disturbances in gait and spontaneous locomotion and exploration in mice

David F. Wozniak[1,4]*, Pamela Valnegri[2]*, Joshua T. Dearborn[3], Stephen C. Fowler[5] & Azad Bonni[2†]

[1]Department of Psychiatry, Washington University School of Medicine, St. Louis, MO 63110, USA

[2]Department of Neuroscience, Washington University School of Medicine, St. Louis, MO 63110, USA

[3]Department of Internal Medicine, Washington University School of Medicine, St. Louis, MO 63110, USA

[4]Taylor Family Institute for Innovative Psychiatric Research, Washington University School of Medicine, St. Louis, MO 63110, USA

[5]Department of Pharmacology and Toxicology, University of Kansas, Lawrence, KS 66045, USA

*Equally-contributing authors

[†]Corresponding author: e-mail - bonni@wustl.edu

Supplementary Methods

Force plate actometer/open field (FPA/OFA) procedure: definition of terms and variables

Trotting: “runs” or bouts of trotting refer to a rhythmic sequence of five or more half strides where diagonally opposite feet act in unison to produce alternating stance and swing phases. Trotting is preceded by walking and followed by running (sometimes referred to as galloping). The force-time wave forms suggest that trotting best characterizes the ambulation occurring during FPA/OF testing

Distance traveled: the line integral of successive pairs of line segments calculated every 0.50 s.

Low mobility bout: a measure of the tendency for a mouse to stay in one place. In the current study, a mouse accumulates a count of one low mobility bout for successfully staying within a 1.5 cm radius circle for 5 s.

Distance away from a wall is a measure of thigmotaxis: the degree to which a mouse avoids the center of an open field. In this study, distance from a wall was calculated for trots only. Therefore, in the current study this measure is associated with active locomotion.

Velocity: defined as the distance covered by a trot divided by the duration of that trot, and expressed as cm/s.

Stride length: a measure obtained by dividing the distance (in cm) covered by a trot by the number of strides in that trot for each mouse.

Stride rate (also known as stride frequency): a measure calculated for each trot by dividing the number of half strides by 2 to obtain number of strides and then dividing the number of strides by the duration of the trot to yield stride rate expressed in Hz (Hz is Hertz or cycles/s).

Within-run force range (WRFR): a variable that provides an estimate of the amplitude of the ground reaction forces produced during a trot.

Supplementary Results

Supplementary Table 1. Values of variables derived from the force-time waveform shown in Fig. 4a from a control mouse during each of two test session halves.

Variables							
Run #	# of Half strides	Distance (cm)	Stride Length (cm)	Duration of run (s)	Velocity of run (cm/s)	Stride rate (Hz)	Within run force range (% body weight)
**FIRST HALF of session**							
1	12	29.69	4.95	1.64	18.10	3.66	82
2	8	20.37	5.09	1.18	17.27	3.39	99
3	10	24.30	4.86	1.45	16.76	3.45	104
4	7	15.94	4.55	1.04	15.33	3.37	101
5	8	20.76	5.19	0.97	21.40	4.12	146
6	5	11.91	4.77	0.64	18.62	3.91	111
7	12	26.81	4.47	1.36	19.72	4.41	111
8	11	26.49	4.82	1.51	17.54	3.64	87
9	8	18.74	4.69	0.96	19.52	4.17	141
10	8	9.87	2.47	0.86	11.48	4.65	76
**SECOND HALF of session**							
1	8	19.78	4.94	0.85	23.27	4.71	155
2	6	15.76	5.25	0.79	19.96	3.80	114
3	5	13.46	5.38	0.62	21.70	4.03	109
4	8	17.04	4.26	1.03	16.54	3.88	108
5	7	19.49	5.57	0.75	25.99	4.67	175
6	6	16.18	5.39	0.75	21.58	4.00	124
7	13	34.26	5.27	1.35	25.38	4.82	149
8	8	21.72	5.43	0.90	24.13	4.44	131
9	7	17.21	4.92	1.03	16.71	3.40	79
10	12	34.40	5.73	1.22	28.19	4.92	171
